# Paroxysmal atrial fibrillation-like bursts in racehorses: do they affect speed and performance? A case control study

**DOI:** 10.1093/jvimsj/aalag095

**Published:** 2026-05-22

**Authors:** Fe ter Woort, Guillaume Dubois, Stephen O’Connor, Emmanuelle van Erck-Westergren

**Affiliations:** Equine Sports Medicine Practice, Waterloo, Belgium; Arioneo, LIM France, Bordeaux, France; Hong Kong Jockey Club, Department of Veterinary Clinical Services, Sha Tin Equine Hospital, New Territories, Hong Kong; Equine Sports Medicine Practice, Waterloo, Belgium

**Keywords:** arrhythmia, equine, exercise ECG monitoring, heart rate variability, racehorse

## Abstract

**Background:**

Paroxysmal atrial fibrillation (pAF) occurs sporadically in horses and has been implicated in poor performance. Diagnosis in large numbers of horses has previously relied on post-exercise ECGs.

**Hypothesis/Objectives:**

Describe pAF-like bursts detected during exercise and determine their effect on speed, stride, and racing performance.

**Methods:**

Retrospective case control study, evaluating records of exercising ECG consultations referred to a specialist practice to identify horses with pAF-like bursts. Controls were selected from the same population as racehorses with normal exercising ECGs.

**Results:**

Eleven cases with pAF-like bursts and 10 controls with normal exercising ECGs were identified, among 100 ECG consultations. The pAF-like bursts occurred in all horses during peak exercise (60-68 km/h; heart rate, 206-217 beats per minute), with normal ECGs during warm-up and recovery. The duration of pAF-like bursts ranged from 10 to 90 s, returning to normal rhythm in all horses before the end of peak exercise. During the pAF-like bursts, 5 horses decelerated, 3 accelerated, and in 3 horses speed was unaffected. Immediately after the pAF-like bursts, 5 horses slowed down, 4 horses did not change their speed and 2 horses accelerated. Root mean square of successive differences (RMSSD) and standard deviation of normal-to-normal intervals (SDNN) during peak exercise were high in all horses. During recovery, RMSSD was normal and SDNN was high in both the pAF-like burst and control horses. Racing performance after the pAF-like burst was available for 8 horses and 7 did not race successfully.

**Conclusion and clinical importance:**

Paroxysmal atrial fibrillation-like bursts occurred during high-intensity exercise in racehorses and may be missed on post-exercise ECGs. These findings emphasize the value of peak-effort ECGs for detecting transient arrhythmias.

## Introduction

Paroxysmal atrial fibrillation (pAF) occurs sporadically in horses and has been implicated in poor performance.^1^^,^^2^ Although persistent atrial fibrillation has been extensively studied, less is known about pAF, likely because of its sporadic nature.^3^ Given the difficulty in characterizing and studying this intermittent condition, recommendations made regarding the course of action after the diagnosis of pAF in a racehorse (rest period, regulatory restrictions, and predicted future performance) are empirical and imprecise. By definition, pAF resolves spontaneously without treatment intervention within 1-5 days after onset.^3-6^ Currently, in studies evaluating the prevalence and impact of pAF on performance, the diagnosis of pAF is based on detecting atrial fibrillation (AF) on a post-exercise ECG.^1^^,^^2^ The recent development, validation, and application of fitness trackers for horses with good-quality ECG recording capacity during exercise have provided unprecedented access to exercising ECGs in combination with speed and stride characteristics.^7^

Although pAF in horses has been described as lasting several minutes or longer, no minimum duration has been established in the veterinary literature.^1^^,^^3^ In human cardiology, AF on a surface ECG is conventionally defined as ≥ 30 s of irregular rhythm^7^, emphasizing the absence of an analogous time-based criterion in equine medicine. During our routine analysis of exercising ECGs, very short irregular atrial rhythms were observed, consisting of brief bursts of irregularly irregular QRS complexes with morphology similar to the normal QRS complexes. Because these episodes resembled pAF but were substantially shorter than classically reported events, they are referred to here as “pAF-like bursts.” These observations raised questions regarding their nature and potential relevance in athletic horses, thereby motivating our investigation.

Our objective was to describe pAF-like bursts detected during exercise using a fitness tracker designed for horses, and to determine the immediate effect of pAF-like bursts on speed parameters and their impact on subsequent racing performance. An additional objective was to evaluate whether any changes in recovery measures or heart rate variability (HRV) data were present in these horses.

## Materials and methods

In our retrospective case–control study, reports of exercising ECG consultations were evaluated to identify cases of pAF-like bursts as well as control cases. The exercising ECGs were recorded using a previously validated fitness tracker (Equimeter, Arioneo, France)^8^ and submitted by the owner or trainer to a specialist private practice for analysis over a time period of 2 years. Training data (including speed, duration, stride length, and stride frequency) were submitted simultaneously. Informed consent for inclusion in the study was obtained from the owner or trainer at the time of subscribing to the ECG analysis service.

The ECG analysis was performed using Kubios Premium software. The artifact correction filter was set to none, and all R–R interval corrections were performed manually after visual inspection of each ECG tracing. For the purposes of the study, episodes were classified as a pAF-like burst when an irregular rhythm was observed, consisting of a sequence of ≥ 6 QRS complexes with similar morphology to the normal QRS complexes. To ensure consistent classification of arrhythmias, ECG tracings were re-evaluated at the time of data collection. All ECGs were manually reviewed to confirm that the irregular rhythm consisted of at least 6 consecutive beats with a QRS morphology matching the horse’s normal sinus complexes. Because no minimum duration criterion for pAF has been established in horses, episodes were included regardless of their duration, provided these appropriate ECG characteristics were present. These arrhythmias are referred to in our study as pAF-like bursts. All recordings were obtained from single-lead exercising ECGs of good to excellent quality. Control ECGs were selected from the same dataset of exercising racehorses and were required to show a normal rhythm throughout peak exercise. Exercise intensity was matched between cases and controls based on the maximal speed achieved during the recorded session. All horses in both groups were Thoroughbred racehorses in active training. Controls were not explicitly age- or sex-matched, but Thoroughbred racehorses typically compete within a narrow age range (approximately 2-6 years), which limits demographic variability between groups.

One horse with a pAF-like burst had an exercising ECG report in which only screenshots of the tracing could be retrieved at the time of the study; the original ECG file was most likely submitted at presentation but could not be located. This horse was included for descriptive information such as presenting complaint and subsequent performance, but was excluded from analyses requiring ECG-derived variables (heart rate [HR] or HRV).

Once identified, cases and controls were retrospectively evaluated for history and presenting complaint. The ECGs were re-evaluated at the time of data collection for characteristics including HRs at various phases of exercise, the moment of occurrence of the pAF-like burst (warm-up, peak exercise, or recovery), pAF-like burst duration and selected HRV parameters before, during, and after the pAF-like burst and during recovery. The HRV parameters were calculated using the Kubios Premium software and the variables recorded for data analysis were root mean square of successive differences (RMSSD) and standard deviation of normal-to-normal intervals (SDNN). These were calculated over a 30-s window for peak exercise, and a 30-s window, and a 5-min window during the recovery.

Peak exercise was defined as the highest HR plateau on the HR graph. Training data also were re-evaluated at the time of data collection using the fitness tracker’s online platform. This approach allowed for the recording of speed, duration, stride frequency, and stride length before, during, and after the episode of a pAF-like burst.

Racing performance was obtained using the free online information available on the websites of the respective racing jurisdictions. Horses were classified as retired, never raced, or racing. If they were racing, racing performance was recorded for all available races in the 6-12 months after the pAF-like episode, and horses were classified as finishing in the top third, middle third, or bottom third.

If available, other exercising ECGs of the horses with pAF-like bursts recorded within 1 month before or after the pAF-like burst episode were identified and evaluated for the presence of arrhythmias.

## Statistical analysis

To compare case and control data, continuous variables are presented as means and SDs or medians (IQR), as appropriate. The normality of the data was assessed using the Shapiro–Wilk test. Normally distributed variables were compared between groups using independent sample *t*-tests (IST), whereas non-normally distributed variables were compared using the non-parametric Mann–Whitney *U* test (MWU). The Benjamini–Hochberg correction was applied to the computed *P*-values. Magnitude of the effect; (mean difference between groups) and corresponding 95% CIs (CI lower and upper) were calculated. Comparison of horse performance between groups was conducted using the chi-squared test. All statistical analyses were performed using the open-source software RStudio version 2022.07.1-554.

## Results

One hundred exercising racehorse ECGs were evaluated. Horses were located in the United Kingdom, France, Australia, and Hong Kong. Eleven ECG reports with pAF-like bursts were identified in 10 horses. One horse had 2 ECGs with pAF-like bursts recorded 12 days apart. Presenting complaints included poor performance (4), previous history of arrhythmia (2), frequent isolated arrhythmia at rest (1), and poor cardiac recovery as perceived by the trainer (1). The ECGs were recorded during training (6), a qualifying race (3), or an exercise test supervised by a veterinarian (2). Ten control ECGs were included, for which the presenting complaint was poor performance in all 10 cases. Two control ECGs were recorded during exercise tests performed by a veterinarian and 8 were recorded during training sessions.

**Figure 1:**
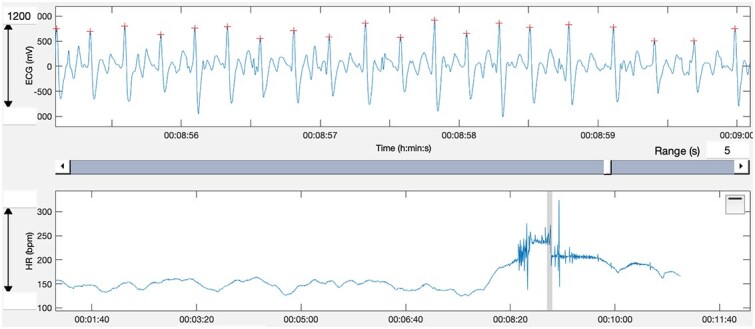
Example of a pAF-like burst recorded during peak exercise. The illustrated segment shows the portion of the ECG in which the rhythm is irregular, with variable R–R intervals and QRS complexes with a morphology similar to the surrounding sinus beats. Short periods of relative regularity may occur within or adjacent to the episode; such regular segments could reflect periods of atrial tachycardia or another supraventricular rhythm rather than pAF. The irregular section displayed here represents the burst included in the analysis. This ECG was recorded using a single-lead fitness-tracker system during high-intensity exercise. Although atrial activity cannot be fully assessed under these conditions, the irregular ventricular response pattern is identifiable. Abbreviation: pAF = paroxysmal atrial fibrillation.

**Figure 2:**
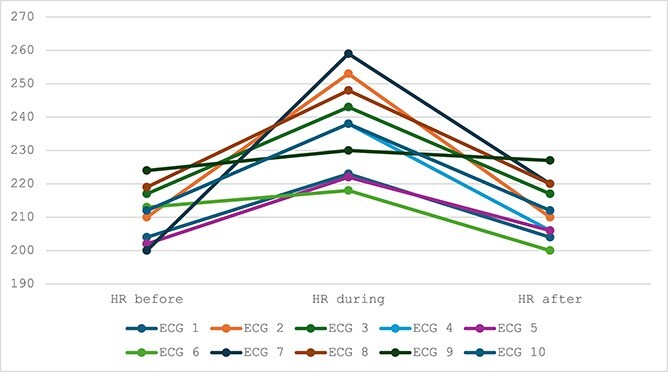
Heart rate (bpm) during peak exercise, before, during, and after the paroxysmal atrial fibrillation-like burst.

The irregular rhythm episodes classified as pAF-like bursts occurred during peak exercise in all ECGs and had durations of approximately 10-90 s (mean ± SD, 22 ± 25 s). These episodes were characterized by an irregularly irregular ventricular response with QRS complexes similar in morphology to the surrounding sinus beats, and each resolved before the end of peak exercise. An example is illustrated in Figure 1. The pAF-like bursts occurred shortly after the onset of peak exercise (*n* = 7), in the middle of peak exercise (*n* = 3), or at the end of peak exercise (*n* = 1). In all horses, the ECG returned to normal before the end of peak exercise and subsequent recovery. The duration of the peak exercise phase ranged from 60 to 210 s, the pAF-like bursts occurred during 8%-50% of the peak exercise duration (mean ± SD 19 ± 14%). During warm-up and recovery, the ECGs were normal. For one of the reports, the ECG could not be found (screen shots of the ECG were included in the report and were used to confirm the pAF-like bursts) but this case was not included in further ECG analysis. Heart rate changes for individual horses are detailed in Figure 2. The mean HR in the 30-s window preceding the pAF-like burst was 211 ± 7 beats per minute (bpm), range 200-224 bpm. The mean HR during the pAF-like burst was 237 ± 14 bpm (range, 228-259 bpm). The mean HR in the 30-s window after the pAF-like burst was 212 ± 9 bpm (range, 200-227 bpm). All horses except for one had an increase in HR during the pAF-like burst and a return to the pre-pAF-like burst HR immediately thereafter. In 1 horse, the pre-pAF-like burst HR was 200 bpm, pAF-like burst HR 259 bpm, and post-pAF-like burst HR was 220 bpm. In this horse, the HR remained higher than the pre-pAF-like burst HR, although the speed was decreasing. In the control horses, mean HR during the peak exercise phase was 215 ± 6 bpm (range, 210-220 bpm).

**Figure 3:**
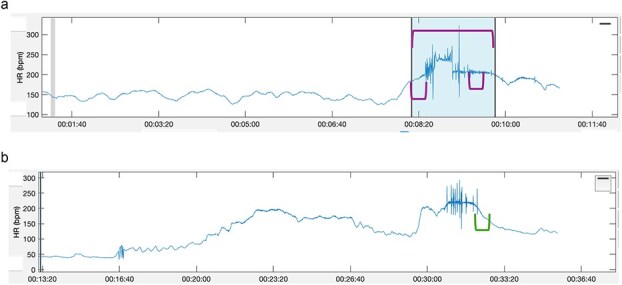
Measurement windows for heart rate variability parameters. (a) Heart rate variability measurement windows during the entirety of peak exercise (duration of the window determined by the length of peak exercise), a 30-s window before the onset of the pAF-like burst and a 30-s window after the pAF-like burst. In this particular horse, there is no sharp decrease in the heart rate because the horse continued to canter after peak exercise and the ECG was removed shortly thereafter. (b) Heart rate variability windows for 5 min and 30 s during recovery, starting at the sharp decrease in heart rate. Abbreviation: pAF = paroxysmal atrial fibrillation.

During the pAF-like burst, the ECG was characterized by an irregularly irregular rhythm, with QRS complexes narrow and similar to the normal QRS complexes during exercise. The HRV parameters and the measurement windows described below are reported and illustrated in Table 1 and Figure 3, respectively. Unsurprisingly, HRV parameters (RMSSD and SDNN) measured during the entire peak exercise period, including the pAF-like burst, were high in all horses when compared to the control ECGs as well as to the published reference ranges in horses exercised on a high-speed treadmill.^8^^,^^9^ When considering a 30-s window during peak exercise before or after the pAF-like burst but excluding the period of irregularity, HRV parameters were normal in all horses. During recovery, in a 5-min time window, RMSSD was normal and SDNN was high in all horses compared with published reference ranges of horses in recovery walking on a treadmill. In a shorter window of 30 s after the onset of recovery, RMSSD was similarly normal and SDNN was high in all horses. However, in all of the control horses, SDNN was also high in the recovery period compared with published reference ranges.

**Table 1:** Heart rate variability parameters of horses during, before, and after the pAF-like burst and during recovery, reported as median (IQR). Individual horse data are provided in the Supplemental material.

**Variable (ms)**	**Phase**	**Cases (pAF-like bursts)**	**Controls**
**RMSSD**	Whole peak exercise	18.0 (12.6-24.3)	5.7 (5.4-8.5)
	Before pAF	4.0 (3.8-14.6)	–
	After pAF	10.5 (6.3-11.8)	–
	Recovery 30 s	4.0 (3.5-5.2)	8.9 (4.2-17.3)
	Recovery 5 min	4.1 (3.7-4.7)	6.3 (4.4-8.5)
**SDNN**	Whole peak exercise	14.9 (13.7-22.0)	6.1 (5.1-9.5)
	Before pAF	8.3 (4.5-15.0)	–
	After pAF	6.2 (4.7-8.4)	–
	Recovery 30 s	27.1 (18.8-41.4)	42.8 (35.9-48.4)
	Recovery 5 min	68.0 (47.1-70.4)	92.5 (68.0-94.8)

Abbreviations: pAF = paroxysmal atrial fibrillation; RMSSD = root mean of successive square differences; SDNN = standard deviation of normal-to-normal intervals.

Training data were available for 10 episodes of pAF-like bursts (1 ECG with missing training data). During peak exercise, mean maximal speed was 62.4 ± 6.2 km/h (range, 42-68 km/h). All ECGs except 2 had a maximal speed > 60 km/h; 2 ECGs were recorded from the same horse with a pAF-like burst occurring at speeds of 42 and 49 km/h. For the control cases, mean maximal speed was 60.3 ± 2.9 km/h (range, 56-64 km/h).

During the pAF-like burst, 5 horses slowed down (decelerating by 2-8 km/h), 3 accelerated (adding 4-11 km/h to their speed) and in 3 horses speed remained unchanged. Immediately after the pAF-like burst, 5 horses slowed down (decelerating by 1-10 km/h), 4 horses maintained their speed and 2 horses accelerated (adding 5 and 7 km/h to their speed, respectively). Mean stride length and stride frequency parameters as well as their respective changes are reported in Table 2. The immediate recovery phase was included for all horses, the longer recovery phase was missing in 2 ECGs where the tracker was removed only 2 min after the end of peak exercise. Recovery strategies were variable, with speeds ranging from 0 to 21 km/h, indicating some horses walked (*n* = 6), some horses trotted (*n* = 3), and 1 horse slowly cantered during its recovery phase.

**Table 2:** Speed, stride length, and stride frequency of horses before, during, and after the short pAF-like bursts. Maximal speed refers to the highest speed achieved during peak exercise. Values are reported as mean ± SD. Individual horse data are provided in Supplementary materials.

**Variable**	**Peak exercise**	**Before pAF**	**During pAF**	**After pAF**
**Max speed (km/h)**	62.4 ± 6.2	–	–	–
**Speed (km/h)**	–	57.1 ± 8.6	57.0 ± 9.1	54.6 ± 8.7
**Stride length (m/stride)**	–	6.62 ± 0.84	6.83 ± 0.77	6.73 ± 0.76
**Stride frequency (strides/s)**	–	2.30 ± 0.17	2.30 ± 0.16	2.25 ± 0.16

Abbreviation: pAF = paroxysmal atrial fibrillation.

After the detection of the pAF-like burst on the exercising ECG, the recommendations included rest (for an undetermined time) and further investigation into the health status of the horse with a clinical examination, blood sample analysis (including cardiac troponin I), bronchoalveolar lavage, and overground endoscopy. The decision to pursue these investigations was at the discretion of the trainer and referring veterinarian, and results of further clinical investigations were unavailable for the majority of the horses.

Five horses had ECGs recorded within the weeks before or after the pAF-like burst. Three horses had had another exercising ECG recorded during training within 2 weeks preceding the pAF-like burst, and all were considered within normal limits. One horse had 2 isolated ventricular premature complexes during peak exercise and the other 2 horses each had one atrial premature complex during recovery. After the pAF-like burst, 2 horses had follow-up ECGs recorded after a 2- to 4-week rest period: the horse with a 2-week rest experienced another pAF-like burst on the follow-up ECG, and the horse with 4 weeks of rest had a normal exercising ECG after its rest period.

Racing performance after the pAF-like burst was available for 8 of 10 horses: 1 horse went on to perform well, finishing in the top 3 within 3 months of the diagnosis, 4 were retired from racing, 2 finished in the bottom third in their subsequent 3 races, and 1 horse never returned to racing. For the control group, performance data were available for 8 horses: 3 horses retired, 1 horse performed poorly (finished in the bottom third of its subsequent races), and 4 horses went on to perform well (finished in the top third of their subsequent races).

The comparison of HR, HRV, and performance variables to the control group and the respective *P* values are reported in Tables 3, and 4a, respectively. The control group exercised at a similar intensity (no differences in maximal speed and peak HR of controls when compared to the peak HR immediately before the pAF-like burst). During the pAF-like burst, the HR was different from that of the control horses. Mean HR for the controls during peak exercise was 215 bpm (range, 210-220 bpm). For the HRV variables, at peak exercise, and after adjustment for multiple comparisons, RMSSD and SDNN were different between groups. These HRV variables during peak exercise immediately before or after the pAF-like burst were not significantly different. During recovery, in a 30-s measurement window, and after adjustment for multiple comparisons, both RMSSD and the SDNN were significantly different between cases and controls. However, the mean value was higher in controls. In the 5-min recovery window, only SDNN remained significantly different, still with a mean value higher in the control horses. Performance was not significantly different in pAF-like cases when compared to controls.

**Table 3:** Comparison of the HR and HRV variables between the group of horses with pAF-like bursts (“case”) and control group using the IST and the MWU. Data are reported as mean ± SD or median (IQR).

**Variables**	**Groups**	**Mean ± SD**	**Median (IQR)**	**Magnitude of effect (95% lower, upper CI)**	***P*-value (Statistical test)**	**Benjamini–Hochberg corrected *P*-value**
**Max HR (bpm)**	Case	237.20 ± 13.86		22.2 (12.2, 32.2)	<.001 (IST)	.001
	Control	215.0 ± 6.01				
**Max speed (km/h)**	Case	62.37 ± 6.21		2.04 (−2.87, 6.96)	.39 (IST)	.46
	Control	60.33 ± 2.91				
**RMSSD whole peak (ms)**	Case	19.25 ± 9.62	17.95 (26.1-11.57)	11.1 (3.93, 18.4)	.002 **(MWU)**	.008
	Control	8.11 ± 5.92	5.7 (9.1-5.3)			
**SDNN whole peak (ms)**	Case	17.32 ± 6.42		9.68 (4.77, 14.6)	0.002 (IST)	.008
	Control	7.63 ± 4.21				
**RMSSD before pAF (ms)**	Case	9.71 ± 9.46	4 (3.6-21.6)	1.61 (−6.03, 9.24)	.32 **(MWU)**	.45
	Control	8.11 ± 5.92	5.7 (9.1-5.3)			
**SDNN before pAF (ms)**	Case	10.77 ± 8.43	8.3 (20.4-3.9)	3.14 (−3.17, 9.44)	.75 **(MWU)**	.81
	Control	7.63 ± 4.21	6.1 (9.9-4.6)			
**RMSSD after pAF (ms)**	Case	9.32 ± 4.06	10.5 (12.5-5)	1.22 (−4.22, 6.66)	.34 **(MWU)**	.45
	Control	8.11 ± 5.92	5.7 (9.1-5.3)			
**SDNN after pAF (ms)**	Case	6.65 ± 3.21	6.2 (10.5-4.3)	−0.97 (−4.94, 2.99)	.82 **(MWU)**	.83
	Control	7.63 ± 4.21	6.1 (9.9-4.6)			
**RMSSD recovery 30 s (ms)**	Case	4.54 ± 1.59	3.95 (5.52-3.42)	−6.39 (−11.7, −1.06)	.04 **(MWU)**	.08
	Control	11.0 ± 7.92	9.0 (17.0-4.0)			
**SDNN recovery 30 s (ms)**	Case	29.95 ± 15.20		−11.9 (−23.4, −0.43)	.04 (IST)	.08
	Control	42.0 ± 10.49				
**RMSSD recovery 5 min (ms)**	Case	4.35 ± 1.16	4.1 (5.25-3.45)	−2.64 (−5.48, 0.19)	.12 **(MWU)**	.20
	Control	7.0 ± 3.84	6.0 (4.0-9.0)			
**SDNN recovery 5 min (ms)**	Case	63.9 ± 15.04		−24.2 (−43.7, −4.67)	.01 (IST)	.03
	Control	88.0 ± 23.19				

Abbreviations: HR = heart rate; HRV = heart rate variability; IST = independent sample *t*-test; MWU = Mann–Whitney *U* test; pAF = paroxysmal atrial fibrillation; RMSSD = root mean of successive square differences; SDNN = standard deviation of normal-to-normal intervals.

**Table 4:** Comparison of horse performance between the group of horses with paroxysmal atrial fibrillation-like bursts (“case”) and the control group. Performance was classified as good (finished in the top 3 of their subsequent races) poor (finished in the bottom 3 of their subsequent races or retired), or retired.

**Variable**	***X*** ^**2**^ **value**	**df**	***P*-value**
**Performance**	1.96	2	.37

**Table 4a:** Comparison of horse performance between the group of horses with paroxysmal atrial fibrillation-like burst (“case”) and the control group. Performance was classified as good or poor/retired.

**Variable**	**X** ^**2**^ **value**	**df**	***P*-value**
**Performance**	1.94	1	.16

## Discussion

Our case–control study identified the occurrence of pAF-like bursts during exercise, which would be missed on a post-exercise ECG. The immediate effect on speed and stride characteristics was variable, but most of these horses did not race successfully after the detection of the pAF-like burst. As expected, HRV parameters were abnormally high during peak exercise including the pAF-like burst, but were normal in the phases recorded immediately before (warm-up) and after (recovery), indicating their limited usefulness in predicting whether a horse will have or has had pAF without the actual ECG recording during the peak phase of a high-speed exercise. Similarly, recovery strategies were too variable among horses to infer any relevant information from measures such as HR after 2 min or other more elaborate recovery parameters. The pAF-like bursts were not consistently detected on other exercising ECGs recorded within a 1-month time frame, further emphasizing the need for repeated ECG monitoring during exercise, especially in cases of poor performance.

The episodes documented in our study were much shorter than the pAF typically described in horses, which usually is reported to last several minutes.^1^^,^^3^ Although the single-lead exercising ECG does not allow definitive confirmation of an atrial origin, the combination of an irregular rhythm and preserved QRS morphology suggests that these bursts arise from supraventricular activity. Their occurrence indicates that very brief irregular rhythm events can appear during intense exercise, and may represent early or transient expressions of the substrate that predisposes some athletes to develop longer episodes of pAF. Even if the mechanism of these short bursts cannot be fully defined here, their detection emphasizes the value of high-intensity exercise ECG monitoring and suggests that precursors or triggers of pAF may be more common than previously recognized. In humans and mice, cardiac remodeling has been shown to occur with exercise, with increased AF susceptibility with increasing exercise dose.^10^^,^^11^^,^^12^ In horses, long-term training increases AF susceptibility.^13^ In addition, cardiac histopathological remodeling also has been detected in athletic compared with sedentary horses, and likely contributes to AF susceptibility.^14^

In this small sample, most horses with a pAF-like burst did not race successfully afterward, but similar outcomes also were seen in some control horses. Both cases and controls were drawn from a population of horses referred because of poor performance or prolonged recovery, which likely influenced subsequent results in both groups. As such, these patterns should be interpreted cautiously and cannot be assumed to reflect a causal relationship. In the control group, performance after the ECG also remained disappointing for 4 of the 8 horses, suggesting that multiple factors unrelated to rhythm may influence subsequent results in racehorses.

Heart rate variability has been used to detect arrhythmias during exercise in horses.^9^^,^^10^ Similar to these studies, the RMSSD and SDNN were high during peak exercise in ECGs with arrhythmias (in cases with pAF-like bursts). The SDNN during the recovery phase was high in these horses with pAF-like bursts (although they were in normal sinus rhythm by then), but this finding was similar in control horses. This situation is likely a reflection of their variable recovery strategies (some walked, some trotted, and some cantered) and as such HR during the recovery, which likely influenced SDNN and should not be compared to published reference ranges of horses in the controlled setting of a treadmill. Indeed, in controls from a similar population of horses with normal ECGs, SDNN was even higher, indicating it is indeed a reflection of the difference in recovery strategies between a field exercise test or training and the treadmill.

Our study had some limitations. One of the main limitations of these ECGs is that they are recorded using a single-lead system. This feature can increase the complexity of the interpretation of the morphology of the QRS complexes in some cases.^8^ As such, the exact origin of the irregular rhythm cannot be precisely determined. However, as defined in the inclusion criteria, the rhythm was irregular, with QRS morphology similar to the normal QRS complexes. An additional limitation inherent to the nature of data collection is the lack of clear follow-up information regarding how much rest each horse received or whether comprehensive poor performance investigations were undertaken, as recommended at the time of the examination and only 2 horses had a follow-up exercising ECG. As such, our study does not permit clear recommendations regarding rest or further investigations considered essential in cases of pAF-like bursts.

In conclusion, our study provides evidence that pAF-like bursts occur during high-intensity exercise in racehorses and can in some cases only be detectable during peak effort. These episodes returned to a normal rhythm before the end of exercise and would be missed on an immediate post-exercise ECG. The arrhythmia was not consistently detected on ECGs recorded before or after the pAF-like burst, further supporting the need for multiple exercising ECG recordings during high-speed training, especially in horses with poor performance.

## Supplementary Material

Supplemental_material_cleaned_up_for_publishing_aalag095

## Off-label antimicrobial declaration

The authors declare no off-label use of antimicrobials.

## Institutional animal care and use committee or other approval declaration

Informed consent for inclusion in this study was obtained from the owner or trainer at the time of subscribing to this ECG analysis service.

## Abbreviations

AF: atrial fibrillation
bpm: beats per minute
HR: heart rate
HRV: heart rate variability
IST: independent sample T-test
MWU: Mann–Whitney U test
pAF: paroxysmal atrial fibrillation
RMSSD: root mean square of successive differences
SDNN: standard deviation of NN (normal-to-normal) intervals
X2: chi-square
