# Supplementary material for: Paroxysmal atrial fibrillation-like bursts in racehorses: do they affect speed and performance? A case control study
Source: J Vet Intern Med. 2026 May 22;40(3):aalag095. doi: 10.1093/jvimsj/aalag095 (PMC13196581; doi:10.1093/jvimsj/aalag095)
Supplement: Supplemental_material_cleaned_up_for_publishing_aalag095 [file supplemental_material_cleaned_up_for_publishing_aalag095.docx]

**S1.** **Heart rate variability parameters of individual horses during, before and after the paroxysmal atrial fibrillation-like burst and during recovery.**

|  | HR peak (bpm) | | | HRV whole peak | | HRV before pAF | | HRV after pAF | | comment | HRV recovery 30 sec | | HRV recovery 5min | |
| --- | --- | --- | --- | --- | --- | --- | --- | --- | --- | --- | --- | --- | --- | --- |
| ECG | HR before | HR during | HR after | RMSSDw | SDNNw | RMSSDb | SDNNb | RMSSDa | SDNNa |  | RMSSD30 | SDNN30 | RMSSD5 | SDNN5 |
| 1 | 204 | 223 | 204 | 18.6 | 14.3 | 3.9 | 3.5 |  |  | pAF at end | 3.7 | 42.8 | 4.7 | 66.9 |
| 2 | 210 | 253 | 210 | 19.1 | 13.5 |  |  | 11 | 6.2 | pAF at beginning | 7.8 | 12.6 | 3.7 | 42.4 |
| 3 | 217 | 243 | 217 | 26.4 | 21.2 | 21.6 | 20.4 | 15 | 10.5 |  | 5.2 | 23.2 |  |  |
| 4 | 212 | 238 | 206 | 26 | 26.6 | 25 | 24.7 | 10.5 | 6.2 |  | 4.2 | 11.3 | 3.2 | 47.1 |
| 5 | 202 | 222 | 206 | 14.2 | 9.7 | 7.5 | 8.3 | 7.5 | 4.3 |  | 5 | 51 | 6.6 | 70 |
| 6 | 213 | 218 | 200 | 10.1 | 22.3 | 3.6 | 5.1 |  |  | pAF at end | 6.5 | 31 | 5.8 | 76.5 |
| 7 | 200 | 259 | 220 | 8.3 | 14.9 | 4 | 3.9 | 3.8 | 5.1 |  | 3.2 | 37 | 3.7 | 68 |
| 8 | 219 | 248 | 220 | 12.06 | 9.2 |  |  | 5 | 2.8 | pAF at beginning | 2.8 | 18.4 | 3.2 | 47.1 |
| 9 | 224 | 230 | 227 | 40.5 | 26.6 | 2.4 | 9.5 |  |  | pAF at end | 3.5 | 20 | 4.1 | 70.4 |
| 10 | 212 | 238 | 212 | 17.3 | 14.9 |  |  | 12.5 | 11.5 | pAF at beginning | 3.5 | 52.2 | 4.2 | 86.7 |
|  |  |  |  |  |  |  |  |  |  |  |  |  |  |  |
|  |  |  |  |  |  |  |  |  |  |  |  |  |  |  |
| mean | 211.3 | 237.2 | 212.2 | 19.3 | 17.3 | 9.7 | 10.8 | 9.3 | 6.7 |  | 4.5 | 30 | 4.4 | 63.9 |
| min | 200 | 218 | 200 | 8.3 | 9.2 | 2.4 | 3.5 | 3.8 | 2.8 |  | 2.8 | 11.3 | 3.2 | 42.4 |
| max | 224 | 259 | 227 | 40.5 | 26.6 | 25 | 24.7 | 15 | 11.5 |  | 7.8 | 52.2 | 6.6 | 86.7 |

This table presents heart rate (HR) and heart rate variability (HRV) parameters of individual horses during peak exercise and recovery. During peak exercise the HR was recorded before, during and after the pAF-like burst. The HRV parameters during peak exercise were measured on the whole peak (including the pAF-like burst), before the pAF-like burst and after the pAF-like burst. Cases are left blank when there was no “before” or “after” because the pAF-like burst occurred immediately at the beginning or the end of the peak exercise. During recovery, the HR and HRV were recorded in a 30 second window (RMSSD30/SDNN30) or a 5 minute window (RMSSD5/SDNN5). Note this table contains 10 ECGs since 1 ECG was missing and excluded from the ECG analysis.

**S2.** **Speed, stride length and stride frequency of individual horses before, during and after paroxysmal atrial fibrillation-like burst.**

| ECG | max speed |  | speed before pAF | speed during pAF | difference before-during | speed after pAF | difference during-after |  | stride length before | stride length during | Difference before-during | stride length after | Difference during-after |  | stride frequency before | stride frequency during | Difference before-during | stride frequency after | Difference during-after |
| --- | --- | --- | --- | --- | --- | --- | --- | --- | --- | --- | --- | --- | --- | --- | --- | --- | --- | --- | --- |
|  | km/h |  | km/h | km/h |  | km/h |  |  | m/stride | m/stride |  | m/stride |  |  | strides/s | strides/s |  | strides/s |  |
| 1 | 61 |  | 50 | 61 | 11 | 51 | -10 |  | 6.3 | 7.2 | 0.9 | 6.5 | -0.7 |  | 2.18 | 2.38 | 0.2 | 2.18 | -0.2 |
| 2 | 68 |  | 67 | 67 | 0 | 67 | 0 |  | 7.2 | 7.2 | 0 | 7.2 | 0 |  | 2.58 | 2.58 | 0 | 2.58 | 0 |
| 3 | 68 |  | 67 | 65 | -2 | 60 | -5 |  | 7.5 | 7.7 | 0.2 | 7.7 | 0 |  | 2.44 | 2.36 | -0.08 | 2.2 | -0.16 |
| 4 | 65 |  | 64 | 61 | -3 | 60 | -1 |  | 7.7 | 7.7 | 0 | 7.6 | -0.1 |  | 2.26 | 2.22 | -0.04 | 2.3 | 0.08 |
| 5 | 60 |  | 56 | 53 | -3 | 60 | 7 |  | 6.5 | 6.5 | 0 | 7.1 | 0.6 |  | 2.34 | 2.28 | -0.06 | 2.34 | 0.06 |
| 6 |  |  |  |  | 0 |  | 0 |  |  |  |  |  |  |  |  |  |  |  |  |
| 7 | 49 |  | 49 | 41 | -8 | 41 | 0 |  | 5.8 | 5.5 | -0.3 | 5.5 | 0 |  | 2.08 | 2.1 | 0.02 | 2.1 | 0 |
| 8 | 42 |  | 42 | 42 | 0 | 42 | 0 |  | 5.8 | 5.8 | 0 | 5.5 | -0.3 |  | 2.06 | 2.06 | 0 | 2.04 | -0.02 |
| 9 | 62 |  | 58 | 63 | 5 | 53 | -10 |  | 6.2 | 7 | 0.8 | 6.5 | -0.5 |  | 2.46 | 2.46 | 0 | 2.28 | -0.18 |
| 10 | 66 |  | 65 | 60 | -5 | 50 | -10 |  | 7.7 | 7.4 | -0.3 | 6.7 | -0.7 |  | 2.3 | 2.2 | -0.1 | 2.1 | -0.1 |
| 11 | 61 |  | 53 | 57 | 4 | 62 | 5 |  | 5.5 | 6.3 | 0.8 | 7 | 0.7 |  | 2.3 | 2.4 | 0.1 | 2.4 | 0 |
|  |  |  |  |  |  |  |  |  |  |  |  |  |  |  |  |  |  |  |  |
| mean | 60.2 |  | 57.1 | 57 |  | 54.6 |  |  | 6.62 | 6.83 |  | 6.73 |  |  | 2.3 | 2.304 |  | 2.252 |  |
| min | 42 |  | 42 | 41 | -8 | 41 | -10 |  | 5.5 | 5.5 |  | 5.5 |  |  | 2.06 | 2.06 |  | 2.04 |  |
| max | 68 |  | 67 | 67 | 11 | 67 | 7 |  | 7.7 | 7.7 |  | 7.7 |  |  | 2.58 | 2.58 |  | 2.58 |  |

This table presents the speed, stride length and stride frequency of individual horses during peak exercise before, during, and after the paroxysmal atrial fibrillation (pAF)-like burst. The maximal speed is recorded over the entire peak exercise. For each parameter, the change (difference) from before the pAF-like burst to during the pAF-like burst and the change (difference) from the parameter during to after the pAF like burst was recorded. Note this table contains 11 ECGs, including the data from the missing ECG for which the ECG report and detailed training data reported in this table were available.
